# MRI in non-pregnancy-related decidualization of deep pelvic endometriosis: a case report and review of the literature

**DOI:** 10.1093/bjrcr/uaag031

**Published:** 2026-07-31

**Authors:** Alessandra Iacono, Beatrice Morelli, Giacomo Avesani, Luca D’Erme, Floriana Mascilini, Anna Fagotti, Evis Sala, Benedetta Gui, Miriam Dolciami

**Affiliations:** Catholic University of the Sacred Heart, 00168 Rome, Italy; Catholic University of the Sacred Heart, 00168 Rome, Italy; Department of Diagnostic Imaging and Radiation Oncology, Fondazione Policlinico Universitario Agostino Gemelli IRCCS, 00168 Rome, Italy; Department of Diagnostic Imaging and Radiation Oncology, Fondazione Policlinico Universitario Agostino Gemelli IRCCS, 00168 Rome, Italy; Department of Women and Child Health and Public Health, Fondazione Policlinico Universitario Agostino Gemelli IRCCS, 00168 Rome, Italy; Catholic University of the Sacred Heart, 00168 Rome, Italy; Department of Women and Child Health and Public Health, Fondazione Policlinico Universitario Agostino Gemelli IRCCS, 00168 Rome, Italy; Catholic University of the Sacred Heart, 00168 Rome, Italy; Department of Diagnostic Imaging and Radiation Oncology, Fondazione Policlinico Universitario Agostino Gemelli IRCCS, 00168 Rome, Italy; Department of Diagnostic Imaging and Radiation Oncology, Fondazione Policlinico Universitario Agostino Gemelli IRCCS, 00168 Rome, Italy; Department of Diagnostic Imaging and Radiation Oncology, Fondazione Policlinico Universitario Agostino Gemelli IRCCS, 00168 Rome, Italy

**Keywords:** magnetic resonance imaging, endometriosis, decidualization, case report

## Abstract

Decidual changes in deep pelvic endometriosis (DPE) are extremely rare. During pregnancy, endometriotic activity and symptoms diminish due to hormonal changes, whereas the endometrial lining undergoes decidualization in response to progesterone. In some cases, a significant hormonal boost can promote the decidualization of endometriotic implants, with endometriomas being the most common sites for such changes. Decidualized endometriosis at sites outside the ovaries is a rarer phenomenon, and the associated imaging features have been studied less. We present a case of non-pregnancy-related decidualization involving multiple sites of DPE. A 33-year-old woman exposed to ovarian stimulation before in vitro fertilization (IVF) presented with chronic pelvic pain and vaginal bleeding. Emergency CT demonstrated irregular solid tissue in the pouch of Douglas and along the bowel loops, raising concern for a neoplastic process. Tumor markers were negative. Transvaginal ultrasound showed a hypoechoic, irregular, hypervascular lesion, and ultrasound-guided biopsy demonstrated decidualized stromal endometriosis. MRI confirmed a solid mass centered in the posterior cul-de-sac with intermediate T2-weighted (W) signal intensity, hemorrhagic foci on fat-suppressed T1W images, marked diffusion restriction, and avid post-contrast enhancement. Similar solid components were also present at multiple pelvic sites, adjacent to DPE implants. Short-interval MRI follow-up showed size reduction; however, due to persistent pain, fertility-sparing surgery was performed, and histology confirmed decidualization of DPE at all sites. This rare case emphasizes that decidualization can also occur on DPE and outside pregnancy, potentially mimicking malignant transformation, highlighting the role of MRI in aiding diagnosis and guiding proper management for these patients.

## Introduction

Endometriosis is a chronic gynecological condition characterized by the growth of functional endometrial tissue outside the uterine cavity, which responds to external hormonal stimuli like the normal endometrium.^1^ Depending on the location and pattern of growth, pelvic endometriosis can be classified into ovarian, superficial, and deep pelvic endometriosis (DPE). Among these various forms, DPE is particularly challenging due to its complex presentation and profound infiltration into pelvic structures.^1^ While ultrasound (US) is usually the first-line imaging technique, MRI represents a reliable and precise tool with high sensitivity and specificity in diagnosing endometriosis, especially for evaluating DPE, providing detailed anatomical assessment, and optimal tissue characterization.^2^

Decidualized endometriosis, a benign condition that occurs in response to some specific hormonal changes, poses additional challenges in both diagnostic and therapeutic scenarios.

Decidualization refers to the transformation of endometrial stromal cells during pregnancy in response to rising progesterone levels, characterized by increased glandular epithelial secretion, glycogen accumulation, and stromal vascularization. This process represents the typical endometrial transformation into decidua, which supports placentation.^3^ Occasionally, endometriotic implants exposed to similar hormonal triggers can undergo decidualization, complicating the clinical picture of endometriosis. Morphological changes in endometriotic implants, such as increased size and vascularity, can make it challenging to differentiate this condition from malignant lesions. The most common site of decidual changes is the endometrioma.^4–8^ Less commonly, decidual changes have been reported in deep endometriosis implants in the pelvis.

## Clinical presentation

A 33-year-old woman who previously received ovarian stimulation before in vitro fertilization (IVF) presented with chronic pelvic pain and vaginal bleeding. Physical examination and laboratory tests yielded no significant results. Pregnancy was ruled out. Abdominal computer tomography (CT) and pelvic magnetic resonance imaging (MRI) conducted in the emergency setting indicated solid tissue in the Douglas pouch and intestinal loops, suspicious for malignancy. Additional laboratory tests were performed at our hospital to evaluate tumor markers, which returned negative results.

## Investigations/imaging findings

A radiological consultation was requested for the MRI images, along with a transvaginal ultrasound (US) and a US-guided biopsy.

Transvaginal ultrasound (US) showed hypoechoic tissue with irregular margins and significant vascularization on color Doppler in Douglas’s pouch, raising suspicion for malignancy.

**Figure 1:**
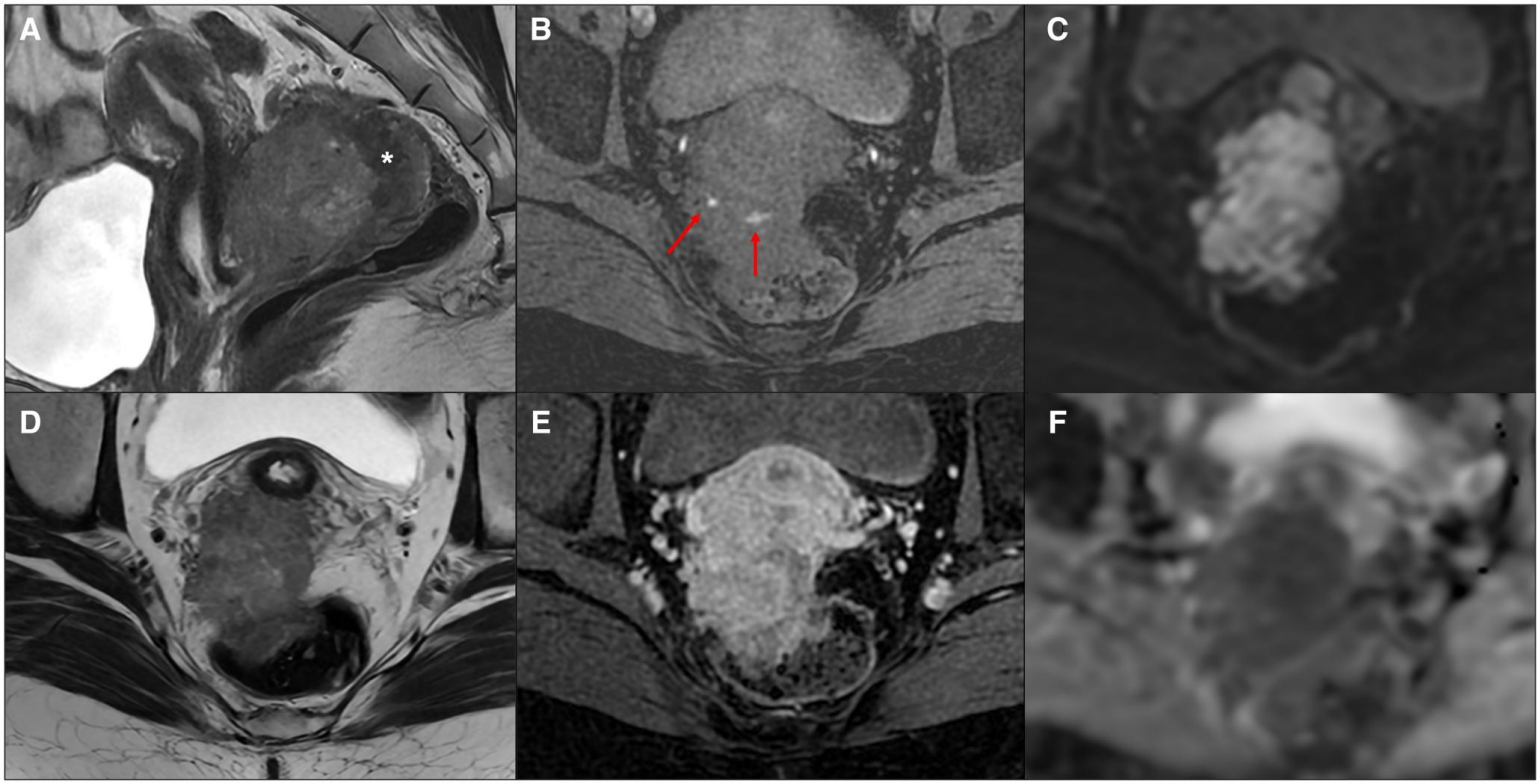
Decidualized DPE in the posterior cul-de-sac. Pelvic MRI demonstrates a solid mass centered in the posterior cul-de-sac, infiltrating the cervix and the rectum. On sagittal and axial T2-weighted images (A and D), the lesion shows intermediate signal intensity, slightly hyperintense relative to the pelvic musculature. The typical *mushroom cap* sign of bowel endometriosis is also visible on the sagittal T2WI (A, star). Fat-suppressed T1-weighted images demonstrate small intralesional hemorrhagic foci (B, arrows). Diffusion-weighted imaging and the corresponding ADC map show marked diffusion restriction (C and F), while post-contrast fat-suppressed T1-weighted images demonstrate avid enhancement of the solid component (E).

**Figure 2:**
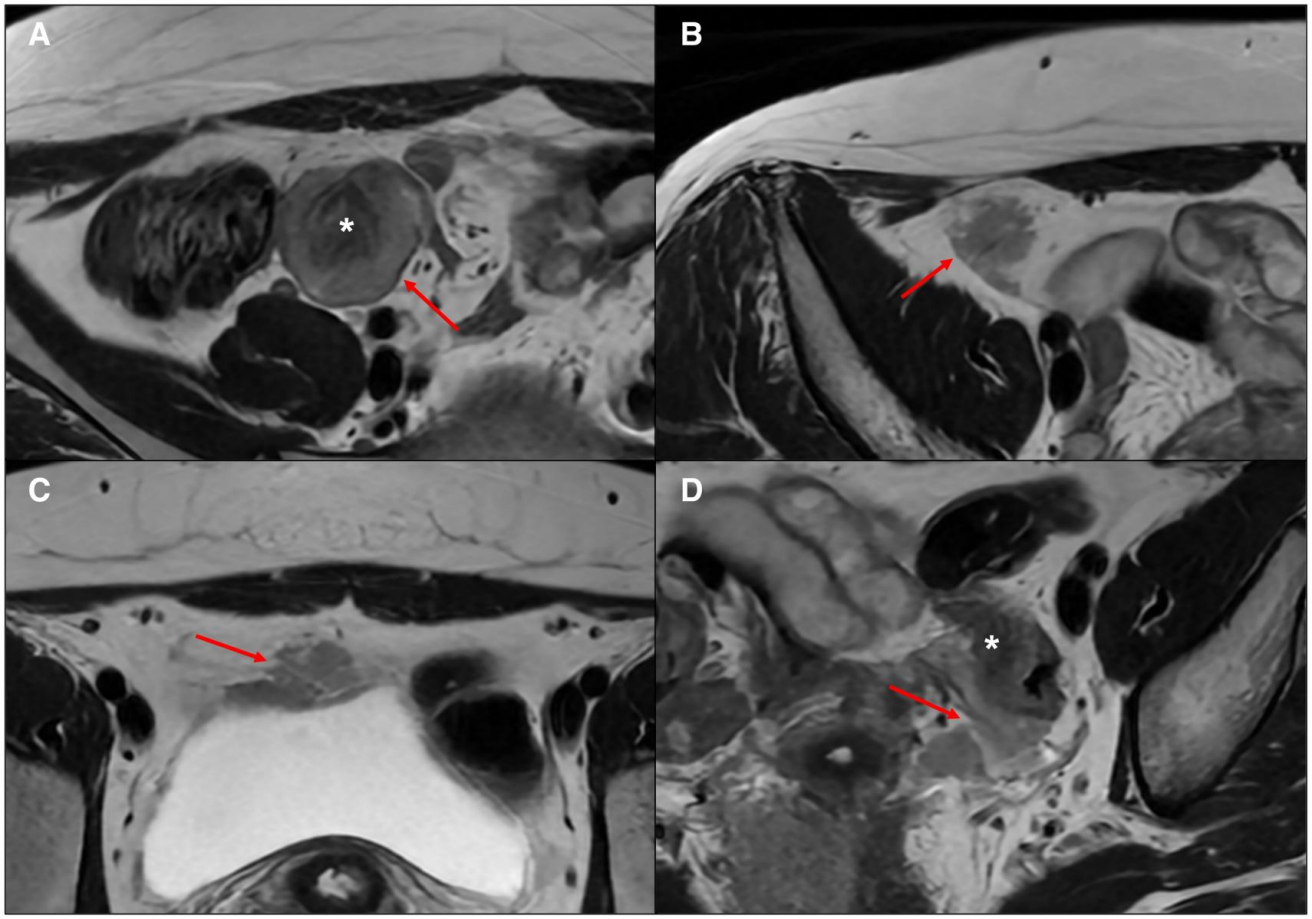
Decidualized DPE at multiple pelvic sites. Axial T2WI images demonstrate additional solid components with similar MRI characteristics at multiple pelvic locations: involving the terminal ileum (A, arrow), adjacent to a bowel endometriosis nodule (star in A); along the right round ligament (B, arrow); in the prevesical space (C, arrow); and between the left parametrium and the mid-sigmoid colon (D, arrow), site of another bowel endometriosis nodule (star in D).

On MRI, we confirm the presence of a solid mass with irregular borders in the posterior cul-de-sac, infiltrating the posterior cervical lip, the rectovaginal septum, and the anterior wall of the middle-upper rectum (Figure 1). The tissue shows an intermediate signal on T2-weighted imaging (WI), isointense/relatively hyperintense to muscle (Figure 1A and D), and small hyperintense foci on fat-saturated (fs)-T1WI, indicating a hematic component (Figure 1B, red arrows), significant hyperintensity on diffusion-weighted imaging (DWI) with low value on the corresponding ADC map (mean ADC value approximately 850 × 10^−3^ mm^2^/s) (Figure 1C and F), highly enhancing on post-contrast imaging (Figure 1E). Where the bowel is infiltrated, a rectal DPE nodule is also observed, with its typical “mushroom cup” appearance on sagittal T2WI (star in Figure 1A). Additional solid tissue components with similar signal characteristics were observed on the terminal ileum (site of a nodule of bowel endometriosis) (Figure 2A), on the right round ligament (Figure 2B), in the prevesical space on the midline (Figure 2C), and between the left parametrium and the mid-sigmoid colon (site of a further nodule of bowel endometriosis) (Figure 2D).

Despite the clinical history of recent ovarian stimulation for IVF—a recognized trigger for decidualization of endometriotic implants—the imaging features alone could not reliably exclude malignancy. The combination of solid pelvic tissue with marked diffusion restriction and avid contrast enhancement, alongside negative tumor markers and clinical background, posed a genuine diagnostic dilemma and warranted histological confirmation. An ultrasound-guided biopsy was therefore performed as a necessary step to achieve a definitive diagnosis prior to any therapeutic decision, particularly given the patient’s young age and desire to preserve fertility. The final histological report indicated decidualized stromal endometriosis.

## Treatment

Given the histological diagnosis of decidualized stromal endometriosis and the patient’s desire to preserve fertility, immediate surgery was deferred, and a short imaging follow-up was advised. An MRI conducted three months later revealed a slight decrease in the size of the solid component in Douglas’s pouch, suggesting a partially self-limiting hormonal process; however, due to persistent, severe, chronic pelvic pain and lack of complete spontaneous resolution, fertility-sparing surgery was ultimately advised.

## Outcome

Definitive histological results confirmed decidualization of deep and bowel endometriosis at all sites.

## Follow-up

The patient is currently stable and in good clinical condition, with mild chronic pelvic pain managed with painkillers; follow-up transvaginal ultrasound reveals thickening of the uterosacral ligament without any additional signs of endometriosis.

## Discussion

Decidualized endometriosis poses a diagnostic challenge due to its rarity and the possibility of being mistaken for malignancy. While US is typically the first-line imaging technique, MRI, by offering superior tissue characterization, can significantly assist in diagnosis.

Decidualization can occur in endometriomas at variable percentages, ranging from 12% to 16% of cases, depending on the study cohort. US can identify changes in decidualized endometriomas, such as rapid dimensional growth, the emergence of solid vascularized components, mainly with papillary morphology rather than septations, and the absence of free fluid.^6^ When MRI is performed, the presence of papillary formations within the endometriomas, with an intermediate T2WI signal and marked DWI restriction, may aid in the diagnosis. Contrast medium (CM) is usually not administered due to frequent decidualization during pregnancy; however, when available, it shows marked enhancement corresponding to the high vascularization observed on ultrasound. Poder et al. described a case in a 33-year-old pregnant patient where serial ultrasounds at 12, 27, and 30 weeks revealed vascularized mural nodules within ovarian endometriosis. MRI demonstrated these nodules as intermediate T2WI signal structures, resembling the decidualized endometrial lining, with restricted diffusion on DWI and ADC mapping.^8^ Some studies have been conducted to evaluate the differential features of decidualized endometriomas in comparison with ovarian cancers. They observed that decidualized endometriomas on MRI show smaller mural solid nodules with higher T2WI signal intensity (similar to normal endometrial decidua) and higher ADC values than malignant tissue, without significant differences in DWI values.^7^

Decidualization of deep pelvic endometriosis (DPE) has been described in a few cases in the literature, and the associated imaging characteristics are much less investigated, mainly with US. Key sonographic features are ill-defined, heterogeneously hypoechoic lesions with a “ground glass” appearance, intracystic excrescences, marked vascular activity, and extremely rapid growth. Moreover, these lesions are typically unilateral and, unlike many malignancies, are not commonly associated with free fluid.^9^^,^^10^ MRI in decidualized DPE provides significant diagnostic support, particularly in identifying solid tissue, which is usually located adjacent to the DPE implants. Decidualized solid tissue exhibits intermediate signal intensity on T2WI, with variable hemorrhagic internal components and restricted diffusion on DWI/ADC map. When CM is administered, as in our case, decidualized tissue reveals marked contrast enhancement. Charkhchi et al. reported a case of a 40-year-old pregnant woman with a finding of a 10 cm pelvic mass during US. On MRI, she presented a large, well-defined, solid mass infiltrating the uterus and the rectum, characterized by T2WI intermediate signal intensity and scattered T1WI hyperintensity foci.^11^ Similarly, Bao et al. described a case of decidualization of bilateral endometriomas and a DPE plaque of the recto-uterine pouch in a 23-year-old pregnant woman. Pelvic MRI without CM confirms three lesions with different features. The tissue in the Douglas pouch shows an isointense signal on T1WI and hyperintensity on T2WI. The left adnexal lesion had hyperintense papillary wall projections on both T1WI and T2WI. The right adnexal lesion was isointense on T1WI and mixed hyperintense on T2WI with restriction on DWI.

In our case, the imaging findings posed a genuine diagnostic dilemma. The solid components showed marked diffusion restriction and avid enhancement, raising concern for malignancy. However, the recent history of ovarian stimulation for IVF, the distribution of the lesions along known sites of deep pelvic endometriosis, and the presence of hemorrhagic foci on fs-T1WI favored decidualization. Despite negative tumor markers and a slight interval decrease in lesion size on follow-up MRI, malignancy could not be confidently excluded. Notably, the mean ADC value (approximately 850 × 10^−3^ mm^2^/s) was lower than those reported for decidualized endometriomas and overlapped with values observed in malignant lesions, further limiting the specificity of diffusion imaging in this setting. The main differential diagnoses were malignant transformation of endometriosis and peritoneal carcinomatosis. Malignant transformation may present with solid enhancing components and restricted diffusion, features that overlap with those of decidualized tissue; however, it is far more commonly described in ovarian than in deep pelvic endometriosis. Peritoneal carcinomatosis may also be considered, particularly in the presence of multifocal solid implants, although the absence of ascites and the negative tumor markers made this diagnosis less likely in our patient. Given the substantial imaging overlap, histological confirmation was required. The slight regression observed at three-month follow-up was consistent with regression of hormonally induced decidualization after cessation of ovarian stimulation, although persistent severe pelvic pain ultimately prompted fertility-sparing surgery.

As far as we know, our case is a rare example of multiple non-ovarian decidualized endometriotic implants occurring outside pregnancy, with contrast-enhanced MRI correlation.

Unlike previously reported cases—which primarily involved decidualized endometriomas in pregnant women^5^^,^^7^^,^^8^ or isolated DPE implants during pregnancy^9–11^—our case highlights that decidualization of DPE at non-ovarian sites can also occur outside pregnancy, triggered by exogenous hormonal stimulation. Awareness of this condition, combined with knowledge of its MRI features, is essential to avoid misdiagnosis as malignancy and to guide appropriate clinical management.

## Learning points

- Decidualization of deep pelvic endometriosis (DPE) is uncommon, mainly occurring during pregnancy but also after strong hormonal stimulation.
- On MRI, decidualized DPE typically appears as a solid component adjacent to DPE implants with intermediate T2 signal, hemorrhagic foci on fs-T1, restricted diffusion, and avid enhancement if contrast agent is administered; multifocal disease may occur if there are additional endometriotic sites.
- Radiologists should be aware of the DPE decidualization pattern, as it can mimic malignant transformation, to raise suspicion and guide further treatment.
